# Hook worm caused chronic anemia found during the procedure of acute gastrointestinal bleeding: a case report

**DOI:** 10.1186/1757-1626-2-105

**Published:** 2009-01-30

**Authors:** Yuan Zhao, Liangjing Wang, Jianmin Si

**Affiliations:** 1 Gastroenterology laboratory, Clinic Medical Research Institution, Sir Run Run Shaw Hospital, Zhejiang University School of Medicine, Hangzhou 310016, PR China

## Abstract

**Background:**

Upper gastrointestinal bleeding with complicated factors is always difficult to find the primary origin.

**Case presentation:**

Here we present a case of a 74-year-old male farmer suffered from acute upper gastrointestinal bleeding caused by gastric ulcer and Mallory-Weiss syndrome and chronic anemia which was at last found caused by hook worm infection.

**Conclusion:**

It tells us that considering multi-possibility when can not explain the symptom with monophyletism is very important for clinicians.

## Background

Upper gastrointestinal bleeding is common in GI department, but the reasons for bleeding are sometimes complicated. We present a case of a male old farmer who suffered from recurrent acute gastrointestinal bleeding with chronic anemia caused by hook worm infection. And this case gives us a lesson to consider multi-reason for bleeding when can't explain the symptom with one etiology. And it also tells us the necessity of recurrent urgent endoscopy.

## Case presentation

A 74-year-old farmer was evaluated for 5 hours of hematemesis and black stools. He denied abdominal pain, or diarrhea, but admitted to take "Fenbid, Indometacin and Metronidazole" because of toothache one day ago. No abnormal past history.

He was afebrile with a pulse of 84 beats per minute, blood pressure of 14.7/9.3 kPa, respirations of 18 breaths per minute. The abdominal exam was normal. His hemoglobin value was 6.0 g/dL (13.9–18.0 g/dL), MCV 70.1 fl (82–92 fl), MCH 21.0 pg (2.0–31.0 pg), MCHC302.2 g/L (310–380 g/L), HCT 0.209(0.37–0.49). OB (++++), liver and renal function and tumor markers were normal.

**Figure 1:**
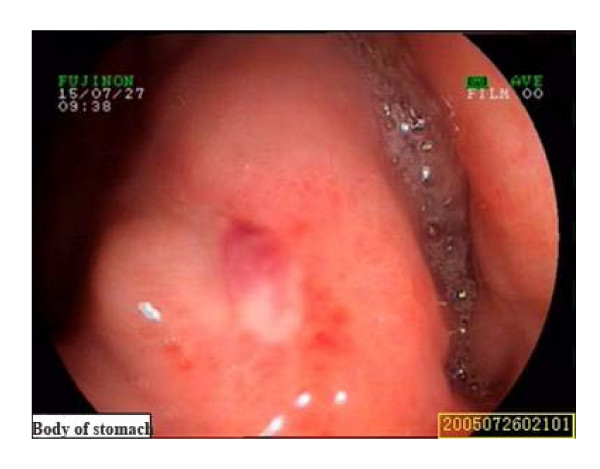
**The Esophagogastroduodenoscopy revealed a stage A1 gastric ulcer with bleeding**.

Urgent upper endoscopy revealed an ulcer in the gastric angle sized 0.4*0.6 cm, and the ulcer was surfaced with white fur and vessel stump, without active bleeding (Fig 1). The patient was stable after fasting, proton pump inhibitor injection, blood transfusion and supportive treatment. But on the morning of the fourth day, the patient felt nausea and vomited about 600 ml fresh blood with clot, with a pulse of 87 beats per minute, blood pressure of 13.9/8.4 kPa, and his hemoglobin value was 5.2 g/dl. Keep the treatment strategy as before. Three days after his condition was stable, the patient was allowed to have fluid. On the tenth day after admission, the patient felt nausea suddenly with hematemesis and melena. The blood pressure at that moment was 8.0/6.9 kPa, and pulse was 100 beats per minute. After 500 ml fresh plasma and 4 units packed red blood cell transfusion, the blood pressure rose back to 12.0/6.9 kPa.

**Figure 2:**
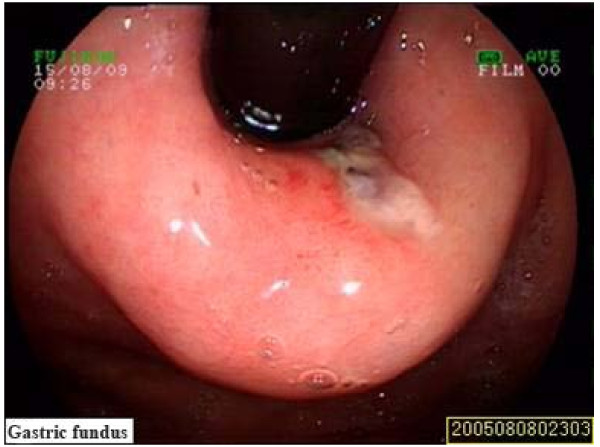
**The Esophagogastroduodenoscopy revealed Mallory-Weiss syndrome**.

**Figure 3:**
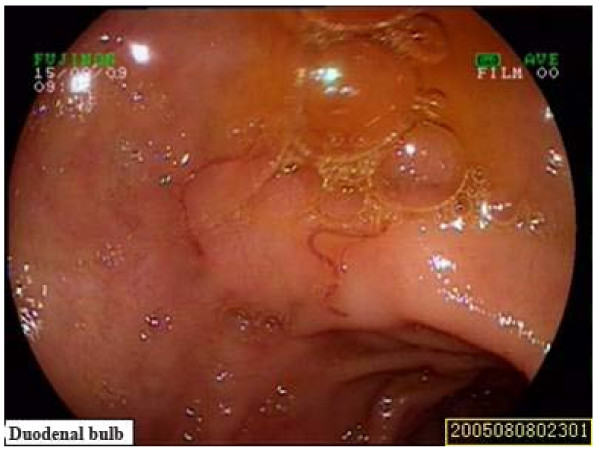
**Hook worm could be seen in the duodenal bulb through the Esophagogastroduodenoscopy**.

Because of recurrent active bleeding and failure of expectant treatment, surgery was considered next step. Upper endoscopy again before surgery found longitudinal laceration with bleeding in the region of the gastro-esophageal junction, and hook warm at the duodenal bulb (Fig 2, 3). After hemostasis treatment under endoscopy and helminthicide treatment, the patient had no further bleeding and hemoglobin value recovered gradually and then was discharged.

## Discussion

Acute upper gastrointestinal bleeding is the most common emergency of GI department. The morbidity is 50–150/10^5 per year according to the BSG guideline 2002, and about 80% can be found the reason for bleeding (Table 1). The achievement ratio of hemostasis is becoming elevated accompanied with fast development of effective medicine and therapeutic methods of hemostasis under endoscopy.

**Table 1:** Etiology of upper gastrointestinal bleeding.

Diagnosis	Percentage (%)
Peptic ulcer	35–50
Erosion of stomach and duodenum	8–15
Esophagitis	5–15
Varicose vein	5–10
Mallory-Weiss Syndrome	15
Malignant disease of the upper gastrointestinal tract	1
Vascular malformation	5
Rare reason	5

Cited from Palmar KR. Guideline Gut 2002

For the patients whose first symptom presented as acute upper gastrointestinal bleeding, but were found chronic microcytic, hypo chromic anemia, it was especially important to consider multi-reason possibility. In this case, chronic blood loss caused by hook warm infection could be the reasonable explanation of unmatchable small volume of hemorrhage at the beginning and heavy grade of microcytic, hypo chromic anemia. The attachment of hookworms' cutting organs to the intestinal mucosa and submucosa and the subsequent rupture of intestinal capillaries and arterioles cause blood loss[1]. Males especially who are involved with agricultural pursuits as the patient in our case are more commonly infected with hookworm than females[2,3]. The patient was diagnosed of upper GI bleeding because of gastric ulcer in the first upper endoscopy. The ulcer might be induced by the medicine he took, but the ulcer was surfaced with white fur without active bleeding when being found. Hematemesis and melena and even shock presented recurrently after expectant treatment. Second endoscopy found Mallory-Weiss syndrome, which was mainly caused by intensive vomiting and retch. And sometimes it can cause fresh blood hematemesis and hemodynamic instability. [4,5]Both the ulcer and Mallory-Weiss with bleeding could be treated through the endoscope. Recurrent urgent upper endoscopy could not only advance the detective rate of etiology for upper gastrointestinal bleeding, but also carry out the hemostasis through it.

## Conclusion

A patient suffered from acute recurrent gastrointestinal bleeding accompanied with chronic anemia gave us a lesson that we should consider multi-reason existing when we could not explain the symptom with monophyletism.

## Abbreviations

GI: Gastro-intestinal; MCV: mean corpuscular volume; MCH: mean corpuscular hemoglobin; MCHC: mean corpuscular hemoglubin concentration; HCT: Hematocrit in blood; OB: occult blood.

## Consent

A written informed consent was obtained from the patient for publication of this case report and accompanying images. A copy of the written consent will be made available on request.

## Competing interests

The above case report was written at Sir Run Run Shaw Hospital. The above mentioned authors have no affiliation to any other institute other than Sir Run Run Shaw Hospital.

## Authors' contributions

YZ, LJW and JMS treated the patent. YZ was responsible for writing the paper and looking up the back ground references. JMS was responsible for over all coordination and final proof reading. All the above mentioned authors read and approved the final manuscript.
